# Spiking Neural Networks in Imaging: A Review and Case Study

**DOI:** 10.3390/s25216747

**Published:** 2025-11-04

**Authors:** Michael Voudaskas, Jack Iain MacLean, Neale A. W. Dutton, Brian D. Stewart, Istvan Gyongy

**Affiliations:** 1 Institute for Integrated Micro and Nano Systems, The University of Edinburgh, Edinburgh EH9 3BF, UK; 2 STMicroelectronics Imaging Division, Edinburgh EH3 5DA, UK

**Keywords:** spiking neural network, Legendre memory unit, neuromorphic engineering, neural network, review, time of flight, imaging

## Abstract

This review examines the state of spiking neural networks (SNNs) for imaging, combining a structured literature survey, a comparative meta-analysis of reported datasets, training strategies, hardware platforms, and applications and a case study on LMU-based depth estimation in direct Time-of-Flight (dToF) imaging. While SNNs demonstrate promise for energy-efficient, event-driven computation, current progress is constrained by reliance on small or custom datasets, ANN-SNN conversion inefficiencies, simulation-based hardware evaluation, and a narrow focus on classification tasks. The analysis highlights scaling trade-offs between accuracy and efficiency, persistent latency bottlenecks, and limited sensor–hardware integration. These findings were synthesised into key challenges and future directions, emphasising benchmarks, hardware-aware training, ecosystem development, and broader application domains.

## 1. Introduction

Spiking neural networks (SNNs) have emerged as one of the most influential paradigms in modern computation, allowing machines to perform complex operations such as pattern recognition, signal processing, and image interpretation with a performance similar to that of humans [1]. Inspired from the human brain, neural networks have evolved over the past few decades into a range of architectures capable of solving high-dimensional problems across several fields, such as medicine, engineering, and security [2]. Specifically in imaging, neural networks have been pivotal in enhancing resolution, segmenting objects, detecting features, and enabling adaptive sensing.

However, most of this progress has been driven by Artificial Neural Networks (ANNs)—dense, frame-based models that operate synchronously and rely on high-precision arithmetic. Although several advances have been made to improve the efficiency of Convolutional Neural Networks (CNNs) [3] and Deep Neural Networks (DNNs), which are common types of ANNs, these architectures still require substantial computational and memory resources [4] and are not well suited for processing temporally sparse or asynchronous data [5]. These constraints make them suboptimal for many real-time or resource-limited applications, particularly in edge processing environments.

SNNs represent the third generation of neural networks (NNs) [6] and have gained increasing attention over the past two decades, unlike ANNs, which have existed since the 1950s. In contrast to their counterparts, SNNs are characterised by event-driven, asynchronous computation [7]. Instead of using continuous activations, they transmit and process information through discrete spike events over time, closely mimicking the behaviour of biological neurons [8]. This sparse, spike-based computation allows SNNs to perform processing only when relevant input is received, resulting in significantly lower power consumption. Moreover, their inherent temporal dynamics align naturally with event- or spike-like neuromorphic imaging systems.

Such systems, like Single-Photon Avalanche Diode (SPAD) arrays or Dynamic Vision Sensors (DVSs), produce outputs not as fixed frames but as event streams or encoded data. Traditional processing methods require these signals to be accumulated, digitised, and post-processed, which introduces latency and inefficiency. On the other hand, SNNs process these signals naturally, potentially enabling end-to-end pipelines in embedded devices.

Despite the potential of SNNs in imaging, research in the field remains unstandardised. There is a wide variability in how spikes are encoded from the sensor’s output, how the network is architected and trained, and how performance is evaluated across platforms. Architectures vary from spiking convolutional models to transformer-inspired networks. Training techniques vary from surrogate gradients to biologically plausible learning rules such as Spike-Time-Dependent Plasticity (STDP). Implementations span from software simulations to custom Application-Specific Integrated Circuits (ASICs) or even neuromorphic processors.

The objective of this review is to analyse SNNs in imaging applications, with an emphasis on system-level integration, training methodology, and hardware deployment. Specifically, this paper aims to achieve the following:

- Compare pre-processing techniques, training approaches, and encoding methods.
- Survey architectures applied to imaging tasks.
- Evaluate implementations on hardware platforms such as accelerators and processors.
- Identify gaps and propose directions for future research.

This paper is structured as follows. Section 2 (Background) provides a background on neuron models and encoding techniques. Section 3 (State of the Art) reviews recent advancements in architectures, training, and hardware. Section 4 (Comparative Analysis) analyses the findings from the reviewed literature. Section 5 (Case Study) presents a case study exploiting the advantages of implementing a spiking architecture consisting of Legendre Memory Units (LMUs) for efficient ranging applications. Section 6 (Challenges and Future Direction) outlines the challenges and future direction of implementing SNNs, and finally, this review ends with Section 7 (Conclusions).

## 2. Background

### 2.1. Brief History

Artificial neurons date back to the 1940s with the McCulloch–Pitts model [9], which was further developed in the 1950s and 1960s with the perceptron and simple feed-forward networks [10]. These models laid the foundation for modern ANNs, which have since evolved into powerful tools across many applications, particularly following the re-emergence of deep learning in the 2010s [1].

Nevertheless, ANNs process data in a dense, frame-based manner, which contrasts with the sparse, asynchronous signalling of biological neurons. This distinction motivated the development of neuromorphic neural networks—most notably the SNN, conceptualised in the late 1990s [6]. A precursor to this development was the work of Thorpe and Imbert [11], who explored biologically inspired models of fast visual information processing. Their emphasis on temporal coding and spike timing helped lay the theoretical foundation for later SNN research.

As technology progressed, SNNs found applications in biologically plausible brain simulations [12] and low-power neuromorphic hardware. Notable examples include IBM’s TrueNorth chip [13] and the SpiNNaker developed at the University of Manchester [14]. These developments enabled large-scale deployment of spiking neurons for sensing and computation.

### 2.2. Neuron Models

Neurons are the basic computational units of biological and artificial networks. This section provides a summary of different neuron models, ranging from biologically plausible versions to computationally efficient models used in neural networks.

#### 2.2.1. Biological Neurons

Biological neurons communicate using brief voltage spikes known as action potentials, which are generated when the membrane potential exceeds a threshold. The classical model, introduced by Hodgkin and Huxley in 1952 [15], provides a detailed mathematical description of the ionic currents responsible for spike generation. While highly accurate, this model is computationally expensive due to its system of non-linear differential equations, limiting its practicality in large-scale simulations.

To address this, the Izhikevich model offers a simplified alternative that captures key dynamical behaviours such as bursting, adaptation, and rebound spiking with minimal computational overhead [16]. Although still biologically inspired, it is more feasible for use in large networks and has been applied in both neuroscience and spiking computational systems [16,17].

#### 2.2.2. Artificial Neurons

**Figure 1:**
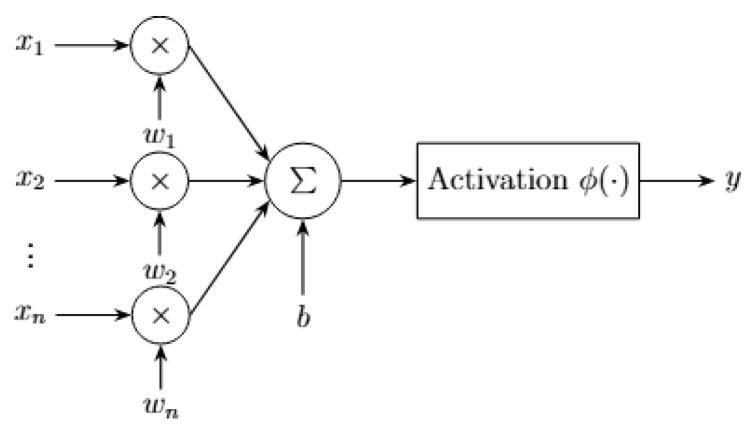
Artificial neuron model with weighted inputs, summation, bias, and activation function.

Artificial neurons, as used in ANNs simplify the biological mechanisms to enable efficient computation. Each neuron computes a weighted sum of its inputs and a bias followed by a non-linear activation function, such as Rectified Linear Unit (ReLU), sigmoid, or tanh [18]. This is illustrated in Figure 1.

Although effective in a variety of vision and pattern recognition tasks, artificial neurons lack intrinsic temporal dynamics. Their design is well suited to modern deep learning hardware but diverges significantly from the behaviour of biological neurons.

#### 2.2.3. Spiking Neurons

Spiking neurons offer a middle ground between biological plausibility and computational efficiency. Unlike artificial neurons, which rely on continuous-valued activations, spiking neurons encode information as discrete events (spikes) distributed over time. This enables them to process sparse, temporal data in an event-driven configuration.

**Figure 2:**
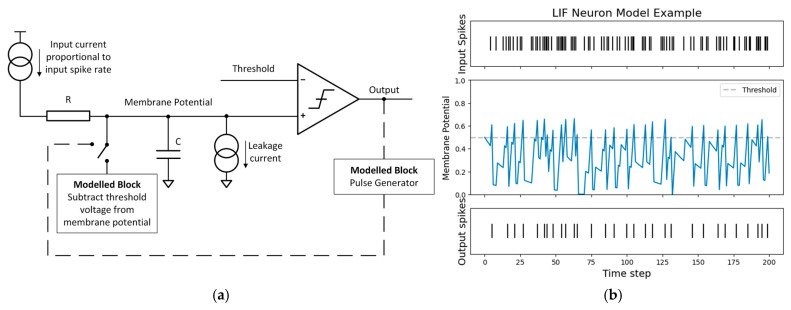
LIF RC model and its simulation: (**a**) LIF RC model; (**b**) LIF simulation. Code adapted from [20].

The most widely used spiking model is the Leaky Integrate and Fire (LIF) neuron. It was originally inspired by the work of Louis Lapicque in 1907, who demonstrated that injecting a short electrical pulse into a frog’s nerves caused a twitch [19]. From his findings, it was later proposed that neurons behave like electrical Resistor–Capacitor (RC) circuits, accumulating charge over time and emitting a signal once a threshold is reached. An example of such an RC circuit is shown in Figure 2a.

Similarly, the LIF model integrates incoming spikes into a membrane potential, which decays over time. When this potential exceeds a threshold, the neuron emits a spike and subsequently resets. A simulation of this process is illustrated in Figure 2b. Simpler variants, such as the Integrate and Fire (IF) model, removes the leak component, while more complex functions—such as the synaptic model, the recurrent model, and the Spike Response Model (SRM) [20]—introduce additional parameters and internal dynamics to better approximate temporal and synaptic behaviours.

Spiking neuron models are particularly well-suited to imaging tasks that involve sparse and asynchronous data, such as those produced by DVSs or SPADs. Their asynchronous and low-power operation makes them ideal candidates for embedded and neuromorphic systems, where energy efficiency and latency are critical constraints.

### 2.3. Spike Encoding

Spike encoding refers to the process of transforming data into sequences of discrete spikes suitable for processing by SNNs. In biological systems, sensory information is naturally transduced into signals by receptor cells; in artificial systems, an explicit encoding stage is often required. The choice of encoding scheme affects both the efficiency and the accuracy of the subsequent processing.

#### 2.3.1. Rate and Population Coding

Rate coding is one of the earlier discovered mechanisms describing how sensory neurons respond to a stimulus. In 1926, Adrian and Zotterman [21] demonstrated that neurons modulate their firing rate in proportion to stimulus magnitude. In an SNN, a continuous-valued input, such as pixel intensity, can be mapped directly to a spike rate or firing probability [22].

Rate coding is straightforward to implement and robust to noise, making it popular in both hardware and software SNN models. However, it does not exploit temporal information and often requires longer observation windows to achieve an accurate representation [23]. In imaging applications, a common use of rate coding is to convert static frame-based data into spike trains, enabling standard datasets to be processed by SNNs [22].

Population coding extends rate coding by representing information across the activity patterns of multiple neurons rather than a single neuron’s firing rate. This redundancy improves robustness to single neuron spike counts and allows encoding of more complex or multidimensional features [20,22].

#### 2.3.2. Temporal Coding

Temporal coding uses the precise timing of spikes to represent information from a stimulus. Common approaches include latency coding, delta-modulation, and Inter-Spike Interval (ISI) coding [20,22].

Compared to rate coding, temporal codes can be more processing-efficient for the same stimulus due to their sparse nature. This makes them particularly well suited to event-driven systems. However, they can also be more sensitive to timing jitter and hardware variability, requiring high temporal precision in both sensing and processing.

**Figure 3:**
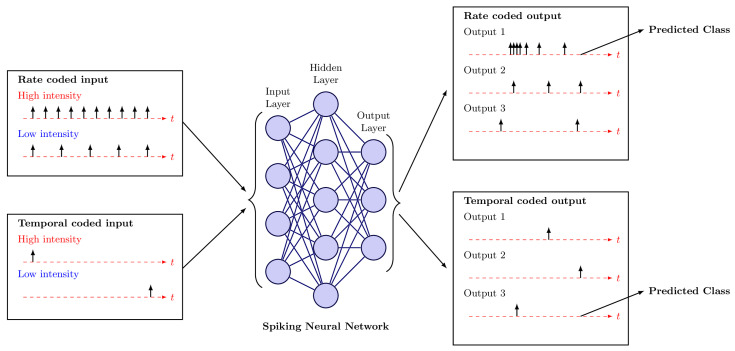
Examples of input spike encoding for high and low intensities in rate and temporal coding schemes. At the output, the neuron with the highest firing rate (rate encoding) or the neuron that fires first (temporal encoding) is the predicted class. The vertical arrows represent spikes.

An example of input and output coding techniques is shown in Figure 3. In rate coding, high stimulus intensity is encoded as a high spike rate, whereas in temporal coding, higher intensity causes a neuron to fire earlier. At the output stage, a rate-coded classifier predicts the class with the highest firing rate, while a latency-coded classifier selects the class whose neuron fires first.

### 2.4. SNN Image Processing

**Figure 4:**
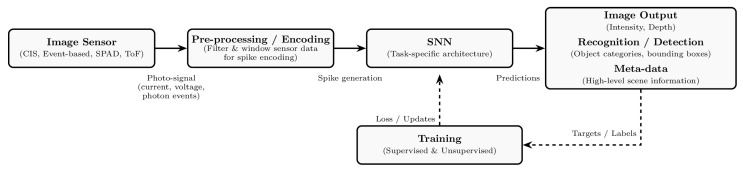
Flowchart of SNN image processing. Sensor data are acquired, pre-processed, and encoded into spikes for the network to process. During training, the predictions are compared with the target outputs, and the parameters are updated accordingly.

The process of handling sensor data using SNNs is illustrated in Figure 4. Raw sensor outputs are first pre-processed and subsequently encoded into spike trains that can be processed by the network. The SNN then generates an output prediction, which may have the form of an image, an inference result, or other high-level scene information.

During training, the predicted outputs are compared with the ground-truth targets, and the resulting loss is used to update the network’s parameters. Training can be performed using either direct learning approaches, such as supervised methods (e.g., surrogate gradient learning) or unsupervised ones (e.g., STDP), or through ANN-SNN conversion techniques, depending on the application and architecture.

Additional details regarding the pre-processing pipeline, architectures, and training methodologies are presented in Section 3.

### 2.5. Summary

This section has introduced the foundations of SNNs and their relevance for imaging applications. Core neuron models were reviewed, from biologically detailed formulations to simplified LIF, along with common neural coding strategies, grouping them into rate- and temporal-based encodings. The general processing pipeline for handling sensor data with SNNs was briefly introduced, outlining the main stages, from sensor input through spike encoding to network output and training. These concepts highlight the distinction between conventional frame-based processing and event-driven computation, where information is conveyed sparsely through spikes in time. Together, these elements establish the motivation for SNNs as an energy-efficient and biologically inspired paradigm for imaging. Building on this foundation, the next chapter surveys the state of the art in SNN algorithms, architectures, and hardware platforms.

## 3. State of the Art

### 3.1. Sensor Integration

#### 3.1.1. Sensor Types

SNN-based imaging systems in the reviewed literature are built mostly on four sensor families: event-based sensors, SPAD, Time-of-Flight (ToF) sensors, and Complementary Metal-Oxide Semiconductor (CMOS) cameras.

The most widely used are event-based sensors, which include DVSs and spike cameras. These devices asynchronously report changes in pixel intensity, providing high temporal resolution and low-latency outputs that align naturally with the event-driven computation of SNNs [24,25,26,27,28,29,30,31,32,33,34,35]. The second group comprises ToF sensors, including Light Detection and Ranging (LiDAR), which output 2D spatial images with depth encoded as a third dimension [36,37,38,39]. The third group includes SPAD arrays, which can detect single photon arrivals, making them highly effective in low-light and time-resolved imaging scenarios [40,41,42,43,44,45]. The final group consists of CMOS Image Sensors (CISs), which produce frame-based or frame-like outputs. In the reviewed works, these are typically used in studies where frame-based imagery is converted into spike trains through pre-processing and encoding steps, enabling compatibility with SNNs [46].

#### 3.1.2. Pre-Processing Techniques

Pre-processing in SNNs encompasses the transformations applied to raw sensor outputs before spike encoding. In the reviewed literature, these can be grouped into three main categories: Temporal Structuring, Event Noise Filtering, and Signal Normalisation and Scaling.

Temporal Structuring was the most common approach, spanning event-based sensors, ToF, and SPAD. Methods included fixed-window binning and voxel grid construction [29,30,37,38], temporal aggregation [35,45], binary histogramming of photon arrival times [42], and threshold-triggered windowing [25]. Variants also adapted spatial structures such as 2D ROI-cropped LiDAR grids with height-based firing rates [39], resolution reduction [27], and high-rate SPAD spike maps [44].

Event Noise Filtering often complements temporal methods. Examples include Address-Event Representation plus thresholding to suppress noise [26], fixed-count accumulation with histogram thresholding for consistent feature density [32], and spatio-temporal filtering with compression and noise subtraction [44].

Signal Normalisation and Scaling appeared in transformer-based, fully connected, and convolutional pipelines, typically to standardise intensity or channel ranges. Examples include image normalisation after random cropping and resizing [47] and scaling within learned spike patch embeddings [48,49]. Table 1 presents a summary of sensor types, their typical pre-processing steps, and representative example operations.

#### 3.1.3. Encoding Methods

Encoding converts pre-processed sensor data into spike trains suitable for spiking computation. The reviewed works employ three main approaches: rate coding, temporal coding, and hybrid encoding, with some architectures embedding spike generation internally without explicit specification.

Rate coding maps stimulus magnitude to spike rate or probability, often using Poisson processes [50] or direct intensity–rate conversion. It appears most frequently in SPAD-based applications, as the method aligns naturally with the sensor’s pulsed output [42,44], where photon arrival counts are directly transformed into firing rates. It was also applied in frame-based recognition tasks [46].

Temporal coding represents information in the precise timing of spikes, including latency and Time-to-First-Spike (TTFS) schemes. It is often paired with event cameras for gesture and activity recognition [25,32], where fixed-count accumulation or threshold-triggered binning ensure spike sparsity and noise reduction. In SPAD-based time-resolved imaging, ref. [42] implemented ISI coding to compress and regularise phase-coded data while preserving timing features, while ref. [41] applied temporally coded spikes to exploit exact timing delays for synaptic weight updates via STDP.

Hybrid encoding combines rate and temporal codes, leveraging their advantages of each scheme. Examples include dynamic scene recognition and mixed-modality sensing [51,52], where firing rate and spike timing were jointly optimised for performance across varying scene dynamics. Moreover, ref. [53] utilised a hybrid method of combining bit-plane coding with rate coding to enhance the performance of the network.

### 3.2. Datasets, Benchmarks, and Applications

The evaluation of spiking neural networks for imaging relies on the interplay between datasets, tasks, and metrics. The choice of dataset strongly constrains the application domain, while the task defines which metrics are appropriate.

#### 3.2.1. Datasets

Early works adapted frame-based benchmarks such as MNIST, CIFAR-10, and ImageNet into spike trains, while neuromorphic datasets such as N-MNIST, CIFAR10-DVS, and DVS128-Gesture enabled evaluation on truly event-based data. For tracking and navigation, KITTI and Sun360 have been used. In parallel, custom datasets support applications in which there is limited availability of data and would be more beneficial from a tailored dataset.

#### 3.2.2. Applications

These datasets underpin a range of imaging tasks. Classification tasks dominate, from MNIST and CIFAR to event-based gesture recognition. Object detection and tracking have been explored on automotive static images, while low-light enhancement and image restoration have been demonstrated using DVS and SPAD data. Each application highlights different strengths of SNNs: low-latency decisions, motion tracking, and noise resilience in low-light enhancement.

#### 3.2.3. Metrics

Evaluation metrics vary by task. Classification and recognition studies primarily report accuracy and F1-score, while detection tasks use mean Average Precision (mAP) and Intersection-over-Union (IoU). Tracking applications assess latency, FPS, and multi-object tracking accuracy. Image reconstruction relies on Peak Signal-to-Noise Ratio (PSNR) and Structural Similarity Index Measure (SSIM). Hardware-oriented studies additionally report energy per inference, latency, parameters, and, rarely, model size.

### 3.3. Spiking Architectures

SNN architectures for imaging sensing tasks follow similar broad structures to conventional ANNs but are adapted to operate on spike-based representations. Across the reviewed literature, three main classes emerge: feed-forward, recurrent, and attention-based. The choice of architecture often aligns with application requirements, balancing computational efficiency, temporal context modelling, and spatio-temporal feature integration.

#### 3.3.1. Feed-Forward Networks

**Figure 5:**
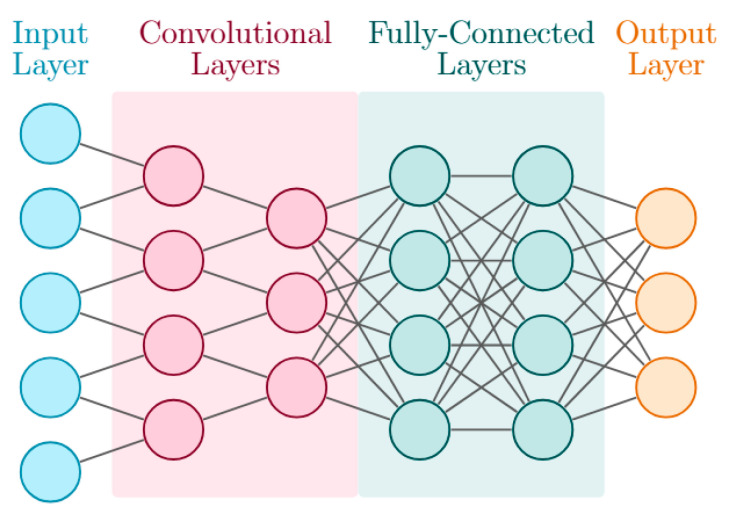
Schematic representation of a CNN architecture, consisting of an input layer, convolutional layers for feature extraction, fully connected layers for high-level reasoning, and an output layer for inference.

Feed-forward networks encompass simple fully connected SNNs, convolutional architectures [26,27,28,29,30,31,33,34,37,38,39,40,42,43,44,45,46,51,52,54,55,56,57,58], and memristive implementations [41,59]. An example of a convolutional architecture is illustrated in Figure 5.

Convolutional layers are often used in the early stages for feature extraction, followed by fully connected layers for inference. Architectures vary from encoder–decoder structures such as spiking U-Nets [29] to residual designs for improved convergence and accuracy [57].

Some works adapt convolutional pipelines to non-frame data, e.g., voxelised LiDAR point clouds [39] or photon-arrival timestamps from SPAD sensors [42], while others optimise for hardware by deploying on Field-Programmable Gate Arrays (FPGAs) [46] or integrating memristive crossbars [41].

This diversity highlights the flexibility of feed-forward SNNs, which remain the dominant choice in imaging tasks due to their simplicity, compatibility with existing deep learning infrastructure, and suitability for low-latency, resource-constrained hardware.

#### 3.3.2. Recurrent Networks

**Figure 6:**
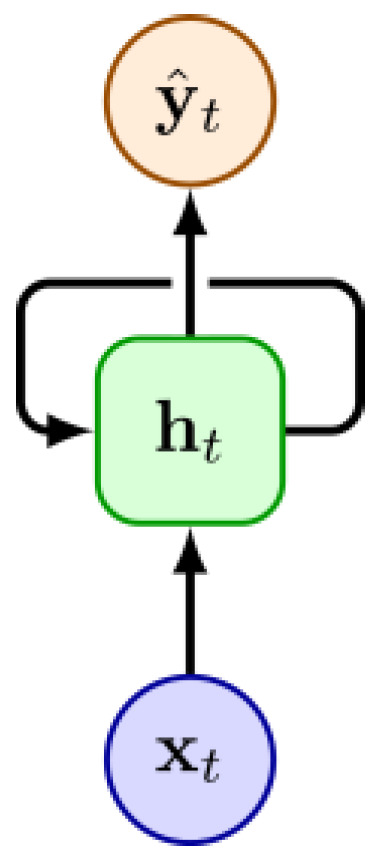
Illustration of an RNN unit, where the input $x_{t}$ is combined with the hidden state $h_{t}$ to predict an output $\hat{y_{t}}$, while the hidden state also feeds back into itself to capture temporal dependencies across time steps.

Recurrent SNN architectures extend temporal processing capabilities beyond the short-term memory of standard feed-forward designs by maintaining hidden states across time steps, making them well suited for sequential and event-based imaging tasks. Figure 6 shows an example of a recurrent unit.

The reviewed works include spiking variants of gated units such as Long Short-Term Memory (LSTM) and Gated Recurrent Unit (GRU) for event-based biomedical imaging [35] and converted low-latency spiking LSTMs designed for efficient hardware deployment [50]. The LMU is another type of recurrent network, which has proven to be effective in encoding temporal dependencies with a modest number of parameters, outperforming other recurrent architectures (e.g., LSTM and GRU) in certain tasks [60]. Reference [36] implements an LMU-based neural network in a dToF application, trying to exploit the network’s ability to process long time series data.

Recurrent models can enhance performance in temporally rich scenarios; however, their adoption in imaging remains limited due to training complexity, increased resource demands, and narrower task specialisation compared to feed-forward designs.

#### 3.3.3. Attention-Based Networks

**Figure 7:**
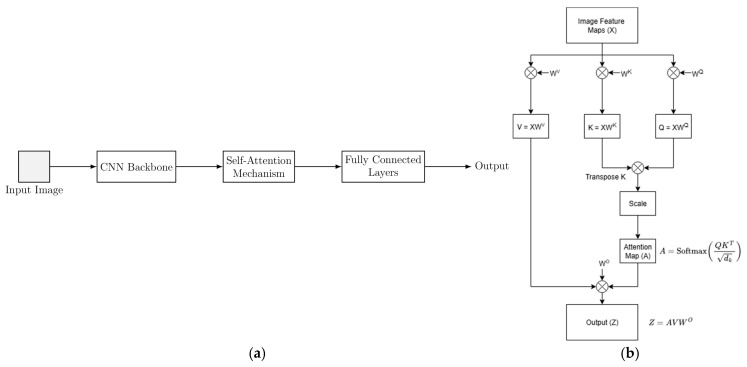
(**a**) High-level example of a hybrid transformer network combining convolutional backbones with an attention mechanism. (**b**) Vanilla self-attention mechanism: input features are projected into Queries (*Q*), Keys (*K*), and Values (*V*), and an attention map assigns higher weight to the most relevant features when combining the values.

Attention-based SNNs adapt transformer principles to the spiking domain, enabling the network to emphasise informative inputs while ignoring less relevant ones [61]. These networks usually result in increased parameters achieving state-of-the-art accuracy in benchmark datasets at the expense of energy efficiency. Unlike convolutions or recurrence, which apply fixed processing patterns, self-attention dynamically re-weights interactions between features across space and time, enabling richer spatio-temporal integration. Figure 7a shows a hybrid transformer architecture, while Figure 7b details the self-attention mechanism. In spiking models, softmax is replaced by spiking neurons, and feed-forward layers are adapted to operate on spike activations.

Early work such as [24] implemented spatio-temporal attention modules to dynamically adjust the neuron firing threshold. Later works, such as [48], replaced layer normalisation and non-linearities with spiking batch normalisation and neurons, introducing spiking self-attention without floating-point multiplications. Source [47] further integrated a residual multi-stage backbone with its Dual-Spike Self-Attention (DSSA) mechanism, enabling efficient handling of multi-scale feature maps and stronger local feature extraction.

The work of [49,62] generalised a transformer-based SNN into a meta-architecture supporting classification, detection, and segmentation. It combines convolutional and transformer-based spiking blocks and introduces multiple spike-driven self-attention operators with linear or sub-quadratic complexity while maintaining sparse addition-only computation for energy efficiency.

Other domain-specific adaptations include [63], which incorporates eye-gaze priors into a spiking transformer for medical image analysis. By guiding spike-form attention maps with gaze information, the model reduces redundant computation and focuses processing on clinically relevant regions, improving both interpretability and efficiency in high-resolution medical imaging tasks.

While convolutional and feed-forward spiking networks dominate imaging tasks, recurrent attention mechanisms have been explored in [64], which integrates a spiking ConvLSTM with a spiking convolutional attention module for event-based gesture recognition.

#### 3.3.4. Neuron and Threshold Variations

Most architectures in the reviewed works employ the standard LIF neuron; nevertheless, several works adapt their own neuron models to improve adaptability, efficiency, or task-specific performance. Variants include adaptive LIF to prevent neuron inactivity [37], homeostasis-enabled neurons with dynamically reconfigurable integration capacitance [41], and adaptive membrane potential neurons that adjust the membrane time constant in real time based on scene motion [29]. Other approaches modify the computational kernel, such as the SRM [31], for flexible postsynaptic and refractory dynamics, signed neurons for imbalanced thresholding similar to leaky ReLU [55], parametric LIF with learnable time-constants [33], and few-spikes LIF for energy-efficient ANN-to-SNN conversion [58].

Threshold control mechanisms appear both independently and in conjunction with custom neuron models. These include attention-guided dynamic threshold adjustment [24], imbalanced thresholds to support negative activations [55], and per-layer learnable thresholds optimised during training [34]. Such modifications, whether applied to the intrinsic neuron dynamics or to its firing threshold, aim to improve temporal precision, robustness to input variation, and energy efficiency.

#### 3.3.5. Beyond Binary Spikes

Conventional spiking neurons are binary, emitting at most one spike per timestep. While energy-efficient, this discretisation limits representational capacity and forces long simulation windows in vision tasks. Recent work explores multi-bit neuron models that encode more information per event, increasing representational accuracy.

Graded spikes process continuous or high-bit amplitudes, as seen in Intel’s Loihi 2 processor [65]. Multi-bit neurons extend the spike space to discrete levels (e.g., ternary outputs [66], multi-threshold firing [67,68], or discrete multi-bit mechanisms [56]), improving accuracy and reducing latency while retaining event-driven computation. Finally, multi-level threshold models such [69,70,71] equip neurons with multiple firing thresholds, enabling richer rate codes that reach near-ANN performance.

### 3.4. Training SNNs

Training strategies in spiking neural networks can be broadly categorised into conversion from conventional ANNs, direct optimisation in the spiking domain, and, less commonly, hybrid approaches.

#### 3.4.1. ANN-SNN Conversion

A widely adopted strategy for training spiking networks is to leverage pre-trained ANNs and convert them into functionally equivalent SNNs. Early pipelines established the feasibility of mapping trained CNNs and Multi-layer Perceptrons (MLPs) into the spiking domain by balancing weights, thresholds, and firing rates [72,73,74]. Subsequent works refined this process, providing theoretical guarantees for threshold balancing and latency reduction [75,76,77]. While these approaches demonstrated competitive accuracy on datasets such as CIFAR-10 and ImageNet, they typically required long simulation windows and elevated spike counts to accurately approximate continuous activations.

Recent works have focused on reducing the spike rate and improving efficiency. Stöckl et al. proposed the few-spikes LIF neuron to emulate ANN activations with only one or two spikes per neuron [58], while Xiao et al. introduced multi-bit spiking to mitigate information loss and support ultra-low time steps [56]. Kim et al. extended conversion beyond classification with Spiking-YOLO, introducing signed neurons and channel-wise normalisation to preserve regression accuracy in object detection [55]. Domain-specific pipelines have also emerged, including SPAD-based temporal imaging [42,43] and LiDAR lane segmentation [39], highlighting the adaptability of conversion strategies to diverse sensing modalities.

#### 3.4.2. Supervised Direct Training

Recent SNN progress relies on supervised surrogate-gradient back-propagation techniques with task-specific losses [78]. Learnable neuron dynamics and learnable thresholds optimise layer-wise firing thresholds and demonstrate state-of-the-art results across event-based classification and object detection, with both CNN and vision-transformer-style backbones [34]. Spiking transformers further extend direct training to attention models via spiking self-attention, achieving competitive ImageNet/CIFAR accuracy at few time steps [48]. Residual formulations designed specifically for spike dynamics enable very deep, directly trained SNNs by establishing membrane-based shortcuts, significantly scaling depth on CIFAR-10 and ImageNet [57].

Supervised SNNs are also deployed in sensing pipelines. In biomedical vision, directly trained SNNs perform event-based white blood cell classification and neuromorphic cytometry, reporting strong accuracy with reduced computation on event sensors [25,27]. In temporal imaging, deep SNNs reconstruct spike-camera videos efficiently [33], and spiking models process direct-ToF (dToF) signals without time-to-digital converters (TDCs) [36], highlighting hardware-aware benefits of end-to-end training.

Overall, supervised direct training now spans learnable thresholds and residual and transformer backbones at low time steps while remaining compatible with event-driven hardware and sensors.

#### 3.4.3. Unsupervised Direct Training

Early STDP-based systems demonstrated that spiking neurons can learn useful visual features directly from unlabelled images. Masquelier and Thorpe showed that intermediate-level selectivity emerges from natural images using STDP and temporal coding alone [79], and later, Kheradpisheh et al. extended this to a deeper convolutional SNN, improving recognition performance via latency-coded feature learning [80].

Recent works refine these principles with richer coding and stabilisation mechanisms. Wang et al. introduced Activeness, an STDP-compatible code combining spike rate and timing to enhance feature quality [52]. In parallel, Shawkat et al. demonstrated that memristive adaptive neurons with intrinsic homeostasis help maintain consistent firing dynamics, improving training stability in hardware-friendly implementations [41].

#### 3.4.4. Hybrid Training

Recently, hybrid training approaches have begun to rise, combining both conversion with direct fine-tuning in the spiking domain. Rathi et al. introduced a hybrid method that uses a converted SNN for initialisation and then applies STDP to fine-tune it, achieving comparable accuracy with significantly fewer time steps and faster convergence [81]. Datta et al. extended this hybrid strategy to recurrent architectures, converting LSTMs and refining them with surrogate-gradient dynamics [50]. Chen et al. proposed a two-stage pipeline where a CNN is first trained conventionally and then temporally fine-tuned as an SNN [51]. Lin et al. similarly applied a warm-up strategy in SPAD-based temporal imaging [42]. These hybrid pipelines exemplify a powerful middle ground, leveraging mature ANN training while embracing SNN sparsity and low-latency benefits.

### 3.5. Hardware and Accelerators

While most spiking imaging pipelines are validated in simulation on Central Processing Units (CPUs) or Graphics Processing Units (GPUs), there is a growing body of work that demonstrates deployment on dedicated hardware. Such implementations are motivated by the strict requirements of imaging tasks—high temporal resolution, low latency, and limited energy budgets—that make conventional hardware poorly matched to the sparse, event-driven nature of spike processing.

#### 3.5.1. Neuromorphic Processors

Dedicated neuromorphic hardware such as Intel’s Loihi [82], IBM’s TrueNorth [13], and the SpiNNaker [14] platform have been employed to accelerate spiking vision. Massa et al. mapped an ANN-SNN converted network onto Loihi for real-time gesture recognition with a DVS camera, achieving nearly ANN-level accuracy with only 37 cores [30]. Similarly, Kreiser et al. demonstrated the use of on-line learning for adaptive visual perception in a robotic setting [83]. Previously, Shukla et al. deployed a convolutional network for object detection on TrueNorth, obtaining competitive accuracy in aerial car counting at a fraction of the power consumption of a GPU [84], while Patiño-Saucedo et al. implemented deep spiking convolutional neural network (SCNN) on SpiNNaker for image classification [85].

#### 3.5.2. FPGA-Based Accelerators

Reconfigurable hardware offers a middle ground between flexibility and efficiency. Lemaire et al. implemented a hybrid SNN on FPGA for satellite image classification, integrating artificial convolutions with spike inference in a single pipeline [46]. Ju et al. achieved fast, low-power classification with an SCNN [86], while Kakani et al. deployed a deep SCNN trained by spike-based back-propagation for object classification tasks [87]. More recent FPGA demonstrations such as SpikeVision adapted transformer-inspired spiking models for gesture recognition, validated with high accuracy on the DVS128 dataset [32].

#### 3.5.3. ASICs and Integrated Vision Chips

Custom ASIC designs provide the highest efficiency by co-designing sensors and spiking processors. Zhang et al. presented a 1 kFPS on-chip tracker using DVS input [26], while Yang et al. introduced bio-inspired SPAD vision chips that combine front-end image enhancement with end-to-end SNN inference, achieving very high inferences per second even in low-light scenarios [44,45]. Lin et al. integrated per-pixel encoders in a SPAD array to generate spike trains directly from photon arrival times [88].

#### 3.5.4. Memristive Approaches

Beyond ASICs and FPGAs, memristive arrays exploit device physics for Compute-in-Memory (CiM). Shawkat et al. developed a SPAD sensor with a memristive neuromorphic core for on-chip processing [41]. Nowshin et al. proposed a CMOS–memristor hybrid accelerator using ISI coding, achieving microsecond-level inference at low power [59], while Roldan et al. built an experimental SNN with hexagonal boron nitride memristor synapses for image recognition [89].

#### 3.5.5. On-Chip Learning

Most hardware demonstrations focus on inference; several platforms are now supporting learning directly on-chip via local plasticity mechanisms. The most prevalent approach is STDP and its variants. For instance, Intel’s Loihi enables programmable local plasticity rules and has been used for event-based gesture recognition and robotic vision tasks [30,83]. Similarly, memristive SPAD sensor chips implement on-line STDP to adapt photon-spike processing at the device level [41].

Beyond STDP, Kwon et al. propose a supervised on-chip training scheme using gated Schottky diodes, enabling parallel weight updates and achieving high accuracy on the N-MNIST event dataset while approximating back-propagation [90]. Vohra et al. demonstrate a CMOS-only circuit that implements unsupervised STDP learning for pattern classification, showing that on-chip adaptation is feasible with standard fabrication processes [91].

Tian et al. [92] presented a memristor-based spiking Generative Adversarial Network (GAN) trained via reward-modulated STDP, enabling on-chip reinforcement learning without backpropagation. Although this study does involve image sensing directly, it demonstrates the potential of such architectures for dataset augmentation within neuromorphic frameworks.

### 3.6. Summary

This section reviewed the state of the art in SNN for imaging, covering training strategies, architectural innovations, and hardware platforms. ANN-SNN conversion remains a widely adopted approach, enabling reuse of pre-trained models, while direct training with surrogate gradients provides a flexible alternative for optimising spiking dynamics. Hybrid and optimisation-based methods are beginning to emerge, combining elements of both paradigms.

On the hardware side, neuromorphic platforms, ASIC, and FPGA prototypes illustrate the feasibility of spiking computation, alongside early demonstrations of in-memory and sensor-integrated approaches. A summary of the hardware platforms reported in the literature, including common properties such as architecture, number of cores, on-chip memory, and power consumption, is presented in Table 2.

Reported applications are dominated by regression and classification tasks, although initial progress has also been made in gesture recognition, depth estimation, and biomedical sensing.

Together, these strands of work define the current landscape of SNN research for imaging. The following section builds on this foundation with a comparative analysis of reported results, quantifying trends and highlighting the limitations that must be addressed for future progress.

## 4. Comparative Analysis

This section presents a comparative analysis of the surveyed works on SNNs in imaging applications. The structure follows two main parts: first, a data-driven analysis using figures extracted from literature is presented; and second, a general discussion of the limitations and gaps in current designs is given.

### 4.1. Analysis of Reported Results

#### 4.1.1. Data Collection Methodology

The comparative analysis is based on the table constructed from the surveyed literature. The studies were collected through keyword searches (“spiking neural networks”, “imaging”, “vision”, “neuromorphic”) from IEEE Xplore, ResearchGate, ScienceDirect, and Springer. Only papers reporting experimental or simulated results related to imaging tasks (e.g., classification/recognition, reconstruction, segmentation, depth estimation, and detection) were included. Works solely on training algorithms or benchmarking without an imaging focus were excluded. A total of 120 papers were initially collected, and 5 were discarded due to insufficient detail. From the remaining set, 42 studies provided consistent quantitative results suitable for further analysis and comparison. Metrics not reported consistently across the literature were omitted from quantitative plots but are discussed qualitatively in Section 4.2.

For consistency, parameters commonly reported in neuromorphic design were extracted, including the following:

- Hardware platform: CPU/GPU, FPGA, ASIC, or neuromorphic processors.
- Sensor type: SPAD arrays, event-based (DVS and spike cameras), ToF (including LiDAR), or CIS.
- Performance metrics: Accuracy, latency, and energy efficiency where available. In cases where the resolution was not specified, the resolution of the training images was used as a proxy.
- Training method: ANN-SNN conversion, direct training, and datasets used for an application.
- Application simplification: Recognition tasks were grouped under classification, and detection tasks were grouped under classification and regression (bounding box estimation).

Data were grouped and plotted only when values were explicitly reported in multiple studies (e.g., energy efficiency $E_{J/\mathrm{MPx}}$ in J/MPx (megapixel), latency in milliseconds). Energy values reported were normalised as follows:

(1) $$E_{J/\mathrm{MPx}}=\frac{E_{\mathrm{reported}}}{N_{\mathrm{pixels}}}\times 10^{6},$$

where $E_{\mathrm{reported}}$ is the reported energy, and $N_{\mathrm{pixels}}$ is the effective image resolution of the processed input.

It should be noted that reported results are often obtained under different experimental settings (datasets, training methods, measurement methodologies). The figures presented should therefore be interpreted as indicative trends rather than absolute benchmarks. The following subsections provide visualisations of these collected data to highlight cross-paper comparisons.

#### 4.1.2. Model Complexity Trade-Offs

**Figure 8:**
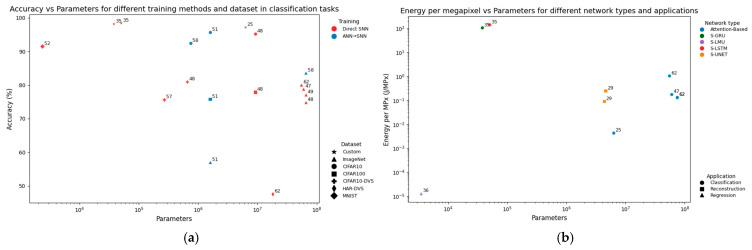
Scatter plots showing how scalability is affected in terms of accuracy and energy efficiency: (**a**) accuracy against parameters for different training methods (colour) and test datasets (shape); (**b**) energy efficiency in J/MPx against parameters for different network types (colour) and applications (shape).

Many studies continue to rely on small or custom datasets. This reliance is visible in Figure 8a, where the highest reported classification accuracies are often tied to custom datasets. While such results demonstrate the feasibility of SNNs for specific tasks, they provide limited evidence of robustness or generalisation to diverse, real-world imaging conditions. For similar datasets, ANN-SNN conversion methods typically achieve higher accuracy at a given parameter count, whereas direct training approaches tend to require larger models to achieve similar performance.

Scalability also emerges as a critical challenge. Energy consumption per megapixel increases linearly with parameter count (Figure 8b), underscoring the inefficiency of large networks. As a result, parameter-heavy models erode the theoretical energy advantages of spiking computation. In contrast, lightweight designs [36] achieve orders-of-magnitude efficiency improvements but often at the expense of accuracy and generalisability. Certain outliers, such as [35], rely on GPU deployment and use energy estimates from earlier studies, which may overstate the reported efficiency.

Together, these results highlight a central relationship in the field: compact networks excel in efficiency but sacrifice accuracy, while large models deliver accuracy but scale poorly. Addressing this trade-off remains a key research priority for enabling practical SNN-based imaging systems.

To further demonstrate the robustness and efficiency of implementing spiking–LMUs, a case study is presented in Section 5.

#### 4.1.3. Deployment Bottlenecks

**Figure 9:**
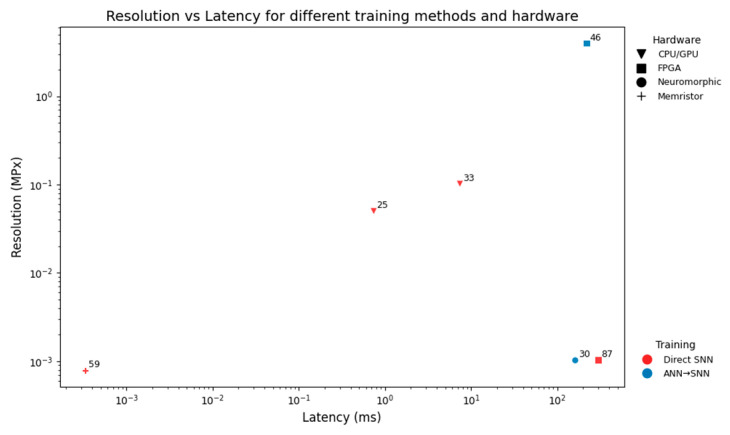
Scatter plot of resolution in MPx against latency in ms. Marker shape represents the hardware platform and colour the training method.

Figure 9 shows that ANN-SNN conversion pipelines consistently incur higher latency than most direct training, even at low resolutions. Directly trained networks therefore appear more suitable for high-resolution, real-time imaging tasks, except [87], which highlights hardware inefficiencies. A memristive CiM implementation [59] achieves the lowest latency, demonstrating the promise of device-level integration, though questions remain about scalability to larger and more complex architectures.

#### 4.1.4. Encoding Suitability

**Figure 10:**
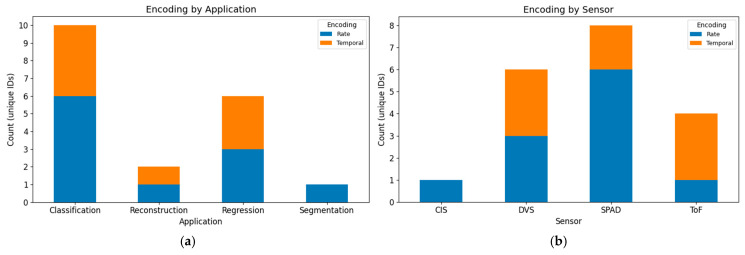
Stacked bar charts showing the distribution of encoding schemes (rate vs. temporal) by (**a**) application and (**b**) sensor type.

To demonstrate the relationship between encoding schemes and both application and sensor type, stacked bar charts are shown in Figure 10. In Figure 10a, both rate and temporal encodings appear across applications, with no single method universally preferred. In Figure 10b, a clearer bias is observed at the sensor level: ToF is consistently paired with temporal encoding, and SPAD devices show a stronger tendency toward rate coding, while DVS sensors employ both schemes.

#### 4.1.5. Application Focus and Sensor Integration

**Figure 11:**
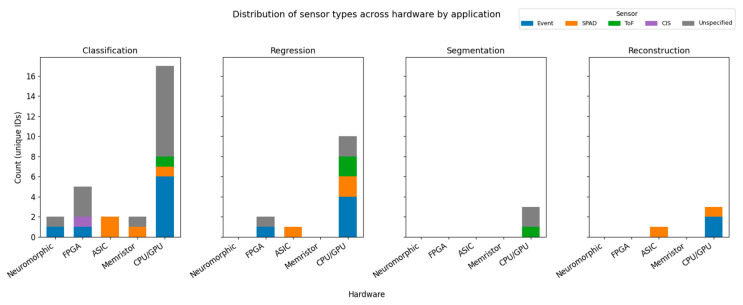
Stacked bar charts showing the sensor distribution for each application across hardware.

Figure 11 illustrates the distribution of different sensor types across hardware platforms for different applications. Classification and regression clearly dominate across all hardware categories, while more complex tasks such as segmentation and reconstruction remain less explored. Notably, image segmentation is the most under-represented application, with no reported hardware implementations to date. The largest bubbles correspond to CPU-/GPU-only studies, underscoring the continued reliance on simulation rather than deployment on dedicated hardware. Moreover, ToF sensors are not implemented on any hardware platform and remain confined to simulation studies, revealing a gap between sensor integration on dedicated hardware.

### 4.2. Limitations of Existing Methods

Building on the above analysis, several limitations in current SNN imaging research can be identified.

#### 4.2.1. Dataset and Generalisation Gaps

A large proportion of works rely on small or highly constrained datasets such as MNIST, CIFAR-10, and DVS-Gesture, with limited exploration of high-resolution, diverse, or real-world imaging data. Cross-dataset evaluation is rare, raising concerns about generalisation and robustness. Applications involving dynamic, high-dimensional, or sparse event streams remain comparatively under-explored. While many works report high accuracy on custom datasets, these results provide limited evidence of transferability, as generalisation beyond the custom domain remains unclear.

#### 4.2.2. Training and Conversion Inefficiencies

Several reported SNNs are derived from ANNs via ANN-SNN conversion. While this approach retains accuracy, it often requires long simulation windows and many time steps, inflating latency and energy consumption. Direct training strategies, though promising, are frequently computationally expensive, slow to converge, and sensitive to hyper-parameter choices. Hybrid approaches and regularisation schemes remain largely experimental and are rarely validated at scale.

#### 4.2.3. Hardware Limitations

Although many works claim hardware relevance, a substantial portion of evaluations remain at the simulation level. Reporting is often partial, limited to estimated energy consumption without corresponding latency or parameter counts. Scaling networks to higher-resolution inputs or deeper architectures typically leads to prohibitive memory footprints and degraded energy efficiency, undermining claims of scalability.

Convolutional and feed-forward attention topologies dominate the field, while attention-based recurrent architectures are almost entirely absent [64]. This stands in contrast to conventional deep learning, where transformers have become the state of the art for vision tasks requiring global context and long-range dependencies. The lack of recurrent attention mechanisms in spiking models limits their applicability to complex imaging objectives. Moreover, the absence of such architectures may also reflect current hardware constraints, as recurrent and attention-based spiking models pose significant challenges for efficient mapping onto existing hardware.

#### 4.2.4. Narrow Application Focus

The literature is strongly biased towards regression and classification tasks, while more complex imaging objectives such as segmentation are under-represented. This narrow scope limits the demonstrated versatility of SNNs for imaging applications and weakens broader applicability.

#### 4.2.5. Energy and Latency Trade-Offs

Energy efficiency is frequently cited as a key advantage of SNNs, yet reported results are often based on estimates rather than measured metrics. Conversion-based approaches accumulate large spike counts and long simulation windows, undermining their efficiency claims. Latency also remains a significant bottleneck, especially for high-resolution inputs or real-time tasks.

#### 4.2.6. Algorithm-Hardware Mismatch

There remains a disconnect between algorithmic design and hardware feasibility. Networks optimised for accuracy are rarely adapted for resource-constrained accelerators, and emerging models (e.g., multi-bit neurons, complex coding schemes, spiking transformers) are not benchmarked on hardware. This highlights the need for co-design methodologies that align sensor innovation, network design, and accelerator development.

#### 4.2.7. Inconsistent Reporting and Benchmarks

The lack of standardised benchmarks for energy, latency, and throughput makes it difficult for direct comparison across studies. Metrics are often incomplete, inconsistent, or measured under incomparable conditions. Without common evaluation frameworks, it is difficult to assess progress and to establish the true advantages of spiking approaches relative to conventional deep learning pipelines.

### 4.3. Summary of Insights

Through all analyses, several repeating issues emerge. First, ANN-SNN conversion inflates latency and energy, whereas direct training achieves lower results but remains under-explored at scale. Second, accuracy and efficiency are often in contrast: compact networks achieve impressive efficiency at the cost of generalisation, while large models deliver accuracy but scale poorly. Third, hardware deployment remains sparse, with most works limited to simulation, minimal sensor integration, and classification applications. Together, these patterns underline the importance of benchmark diversity, hardware-aware training, and co-design spanning sensors, networks, and accelerators.

## 5. Case Study: Spiking Neural Network-Based TDC-Less dToF

### 5.1. Introduction

Combining arrays of SPADs with integrated processing enables solid-state dToF sensors to be built, with the potential of compact, robust, and low-cost 3D sensing solutions for applications ranging from smartphones to robotics and the automotive industry. To manage the high data rates produced by SPADs, architectures with on-chip histogramming have been proposed [94]. However, the associated memory and power requirements limit the scalability of such architectures. Partial histogramming can overcome scalability issues but at the cost of reduced laser power efficiency and increased susceptibility to motion artifacts [95]. Furthermore, as in full histogramming, high-frequency reference clocks are typically used for photon timing, and hence the chip power consumption can still be significant. In recent years, several alternatives to and variants of histogramming have been proposed, including spline-based sketches [96], count-free histograms [97], histogram-free processing of photon time stamps [98], and neural-network-based processing [99].

This study follows-on from [36], where a new single-photon dToF processing scheme was developed using SNNs with LMU architecture [60]. The LMU operates by implementing a memory cell which approximates the unit delay ($y\left(t\right)=u(t-\theta$)) such that any input seen within θ seconds is represented within d memory units and can be approximately reproduced. This behaviour allows for the network to store all the SPAD events over the whole exposure to make its final prediction.

**Figure 12:**
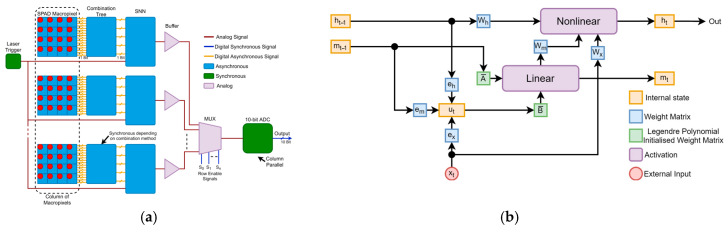
(**a**) Proposed architecture of TDC-less dToF sensor (reproduced from [36]) showing a column of pixels. Each pixel has a 4 × 4 array of SPADs followed by a combination tree (e.g., an asynchronous adder), whose output is presented to an SNN. The outputs of the SNNs are, in turn, fed to a column parallel ADC. (**b**) Block diagram of LMU SNN [60], reproduced from [36].

**Figure 13:**
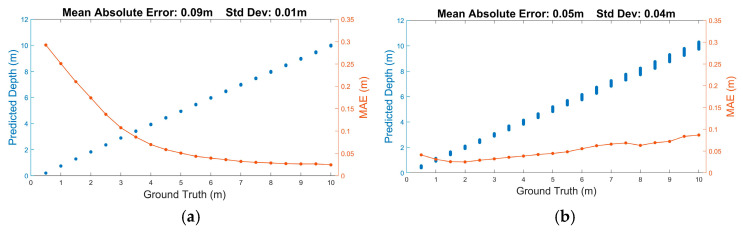
Depth estimation of (**a**) CMM and (**b**) SNN processing based on synthetic photon data. The graphs include scatter plots of estimated depth (blue points) as well as the mean absolute depth error (orange) calculated using repeated simulations at a range of assumed distances. The CMM results assume histogram generation (with bin size 500 ps) prior to depth processing. The following system parameters were assumed: 45 cycles of 940 nm laser source with 4 ns pulse duration and 640 nJ pulse energy. SPADs are taken to be 10 µm in size; an f1.2 objective with a 10 nm bandpass filter is presumed to be in front of the array. The ambient level is 1 klux, and object reflectivity is 40%.

**Figure 14:**
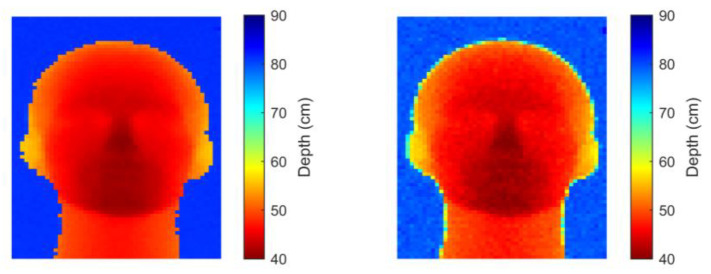
Depth maps generated by applying CMM (**left**) and SNN (**right**) processing to real single-photon LIDAR data from [96], converted into SPAD events. Good correspondence can be seen even though the IRF is non-Gaussian, compared with the Gaussian IRF used in SNN training.

The scheme (shown in Figure 12) benefits from the low-power capabilities of SNNs [100], together with the ability of LMUs to analyse long sequences of time series data. In tests using simulated SPAD data, the scheme is found to have lower precision than conventional (centre-of-mass, CMM) dToF processing on histogram data but higher overall accuracy, successfully learning to compensate for SPAD saturation effects (Figure 13). When run on measured data (Figure 14), the SNN is seen to successfully reproduce the main features of a mannequin’s head, despite having been trained on synthetic data only. In the figures, depth is the distance estimated by each pixel to the object’s surface.

**Figure 15:**
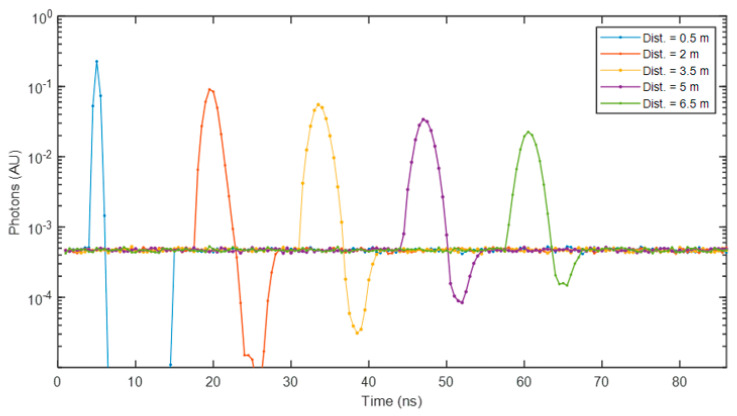
Photon arrival histograms at different target distances, showing the SPAD pile-up effect that compresses peaks into single bins in conventional dToF pipelines. The LMU architecture exploits the recurrent SPAD firing pattern under pile-up to correct for this effect, reducing close-range errors (see Figure 13b).

Figure 15 illustrates histograms of photon time of arrival resulting from the same target at different distances. The histograms were generated using the same synthetic photon dataset as in Figure 13. The plot highlights the SPAD pile-up effect observed at short distances, resulting in histogram compression, where the entire peak may be confined within a single TDC code or histogram bin in a conventional dToF processing pipeline, leading to inaccurate distance estimates. In contrast, the spiking–LMU method effectively recognises and corrects for pile-up, yielding lower errors at close ranges, as demonstrated in Figure 13b. When operating in a pile-up condition, the SPAD fires consistently at approximately the same time in each laser cycle (followed by a dead-time, during which photon detections—whether signal or ambient—are inhibited, leading to the trough in the histogram). It can be conjectured that it is this recurrent SPAD output sequence that the SNN detects and leverages for refining the depth estimate.

### 5.2. Architecture

The memory cell m is implemented using Legendre polynomials in a state space representation, as shown below:

(2) $$\dot{m}\left(t\right)=A\;m\left(t\right)+B\;u(t),$$

where m(t) represents the current state of the memory cell at time t, u(t) is the current input, while *A* and *B* are matrices of the state space representation, which are initialised using Legendre polynomials:

(3) $$\begin{array}{ccc} A={\left[a\right]}_{ij}\in R^{d\times d}, & a_{ij}=\left(2i+1\right), & \left\{\begin{array}{cc} -1 & i<j\\ {\left(-1\right)}^{i-j+1} & i\geq j \end{array}\right. \end{array}$$

(4) $$\begin{array}{ccc} B={\left[b\right]}_{i}\in R^{d\times 1}, & b_{i}=\left(2i+1\right){\left(-1\right)}^{i} & i,j\in [0,d-1] \end{array}$$

where d is the order of the memory cell m. The input signal can be reproduced using the state space equation:

(5) $$y\left(t\right)=C\;m\left(t\right)+D\;u(t)$$

**Figure 16:**
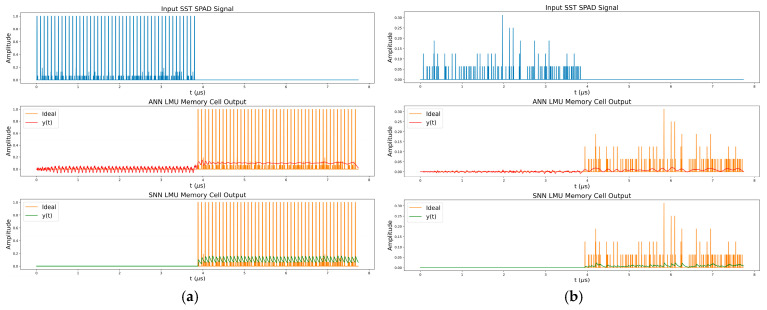
Operation of the 56 ordered memory cell if *u*(*t*) is a series of SPAD events combined using SST with a window size θ of 3.87 µs (corresponding to 45 laser cycles). The cases of (**a**) high signal level and (**b**) lower signal level are shown. The ideal signal recreation of the input signal (blue) using the current state of the memory cell is shown in orange. The approximated recreation using an ANN version of the network is shown in red, while the recreation using an SNN is shown in green. Neither the ANN nor SNN version can make a perfect recreation of the input signal, as they can only store a finite amount of information. The recreation of the input can be improved by increasing the order d of the memory cell at the cost of more neurons and, therefore, an increased demand on power and silicon area.

The *C* matrix needed to reproduce the input signal from θ seconds ago is simply all ones. However, the *C* matrix can be modified to produce outputs for different delays within $0\leq \theta^{\prime }\leq \theta$. The D matrix is always zero. This operation can be visualised using Figure 16, where the response for an ANN and an SNN memory cell of the order 56 stores and then reproduces a SPAD signal, resulting from a synchronous summation technique (SST) [101], for a θ of 3.87 µs.

The LMU network uses this memory cell to store previously seen information in a compressed format. Another ensemble of neurons, the hidden units, can then use the compressed representation to make a prediction based on all the information seen within the last θ seconds. In theory, the SNN makes predictions based on a sliding window of a set number of laser cycles, then once the network reaches a steady state it will be capable of producing a surface depth prediction after every laser cycle.

The network is trained using the Nengo-DL Python package (version 3.6.1) [102], which trains the SNN using surrogate gradient descent based on approximated firing rates of the networks neurons. Nengo-DL uses TensorFlow as the backend, enabling the network to be trained using a GPU, which in this case was an RTX 3090. A total of 12,000 synthetic photon sequences were generated for training and validation (10,000 for training and 2000 for validation). The trained model was then evaluated on 40,000 test sequences, corresponding to 10,000 samples under each of four different ambient and reflectivity conditions. The model was not retrained for subsequent experiments.

### 5.3. Results

**Figure 17:**
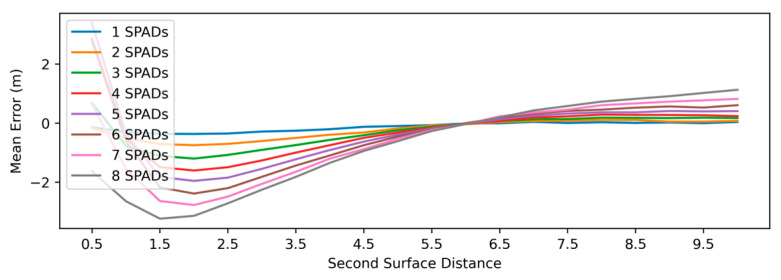
Mean error in SNN depth estimate of primary surface (situated at 6 m distance) in the case of a second surface (at different distances). Each plot corresponds to the secondary surface covering a different number of SPADs (within the 16 SPADs constituting a pixel). Surface reflectivities of 40% and an ambient level of 25 klux are assumed.

**Figure 18:**
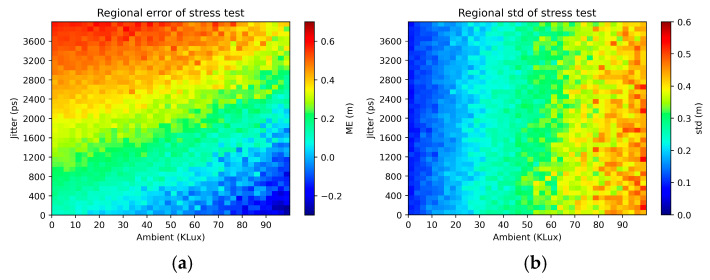
Heat maps of (**a**) the mean error and (**b**) standard deviation of the error for varying levels of SPAD IRF width and ambient level. A target distance of 10 m with surface reflectivity of 40% is assumed.

Figure 17 plots the mean error in the depth estimate in the case when the SPAD macropixel observes two distinct surfaces. The plots indicate that the SNN reports a weighted average of the two surfaces, with the weighting favouring the surface that is closer to the sensor (as expected, due to the higher return signal from the latter). End effects are noted when the second surface is close to the sensor. In Figure 18, heat maps are used to capture, for the single-surface case, the variations in the mean error (Figure 18a) and standard deviation (std) of the error (Figure 18b) as the SPAD IRF and ambient level are increased beyond 100 ps and the maximum of 30 klux that the SNN was trained for. As the IRF is made wider, an increased bias is observed in the depth estimate, although it is somewhat negated by an apparent bias in the opposite direction at high ambient levels. On the other hand, the widened IRF is seen to have a relatively minor effect on the std of the error (quantifying the precision of the depth estimates). Although the std does increase with the ambient level, the increase is seen to be linear. Overall, no sudden, drastic deteriorations in performance can be observed from the graphs.

**Figure 19:**
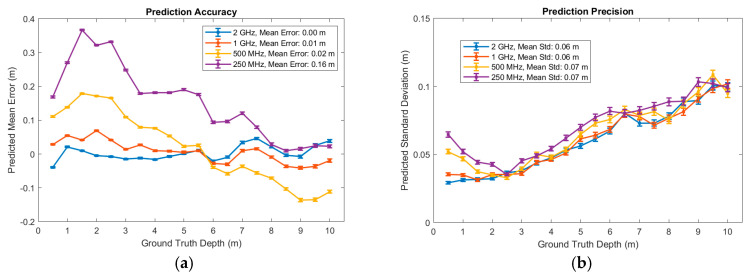
Ranging performance of SNN, based on synthetic data for 70% reflectance and 1 klux ambient light. In this case, an STT is used for SPAD combination, and the sampling frequency is decreased from the nominal 2 GHz used in training. The performance is quantified in terms of (**a**) accuracy and (**b**) precision; the same system parameters are assumed as in Figure 13. In terms of accuracy, only a modest change at 1 GHz is observed, but a marked decrease at lower frequencies is observed (this may be countered by re-training the network). On the other hand, the sampling frequency does not have a significant impact on the depth precision, apart from at close range (possibly due to the SPAD IRF becoming compressed due to SPAD saturation).

In case an STT (rather than an asynchronous adder) is used to combine SPAD events, it becomes useful to assess the impact of reducing the sampling frequency with no retraining. Figure 19a shows a deterioration in accuracy but only a minor decline in precision (Figure 19b).

**Figure 20:**
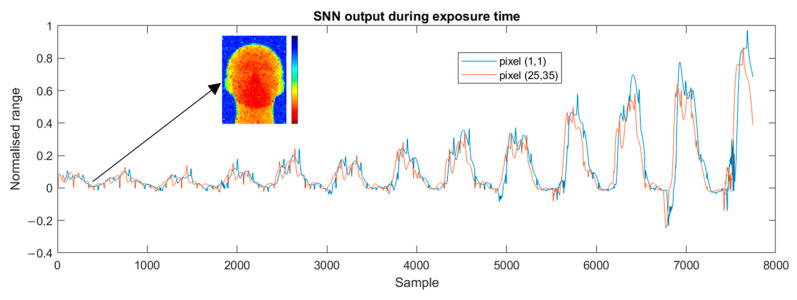
Output of SNN for the same data as in Figure 13. The outputs corresponding to two pixels (one in the background and one in the middle of the head) are shown. Whilst the output is seen to oscillate over the exposure time, reasonable depth maps may be obtained even early on during the exposure (such as at sample 364 as depicted), suggesting that early sampling of the SNN output may be useful in some circumstances.

Figure 20 depicts the evolution in the SNN output during exposure of two pixels in the mannequin data. It is worth noting that a reasonable depth map is produced even early in the exposure, raising the possibility of “early sampling” when a high signal level is sensed.

The power consumption of the SNN is estimated to be in the nW range, assuming typical firing rates seen in simulations, and the generation of depth estimates at video rates [36] (estimated as 203 pJ per inference, 6.09 nW@30 fps with 20 nm [103]). As regards to the area of a potential circuit implementation, it should be noted that compact (0.04 µm^2^) LIF neurons have been proposed in advanced technology nodes (7 nm FinFET) that can fire at 1 GHz rates required in this study [104]. Noting that the SNN presented here has 610 neurons and 3506 synapses, and assuming a synapse size similar to that of neurons, a total circuit area in the region of 164 µm^2^ is estimated, which would fit under a typical 40 × 40 µm SPAD macropixel [94]. The latency of the network is only limited by the ADC’s conversion cycle, as each inference processing is available on photon arrival.

### 5.4. Conclusion

These preliminary results suggest that the SNN is reasonably robust to unfamiliar operating conditions, which bodes well for applications such as a low-power object detection for robots or drones, avoiding the need for re-training, which can be time-consuming. Modifications are being considered to the SNN architecture to enable longer-ranging distances and the detection of multiple surfaces. In addition, adjustments will be explored to the window size and operation to enable faster and more accurate depth prediction after every laser cycle.

## 6. Challenges and Future Direction

As highlighted in the case study, SNNs demonstrate strong potential for low-power embedded applications. However, significant work remains to realise practical, large-scale systems.

Building on the comparative analysis in Section 4, this section zooms out to consider broader challenges and future directions for SNNs, drawing on recent surveys and reviews [105,106,107,108,109,110]. These works collectively highlight persistent bottlenecks and emerging opportunities across algorithms, hardware, sensors, and applications. The discussion is organised around key themes, explicitly linking them to the limitations identified earlier.

### 6.1. Scaling and Ecosystem

Kudithipudi et al. [105] emphasise that neuromorphic computing is at a critical juncture, with the field awaiting its “AlexNet moment”—a demonstration of scale that proves disruptive potential. Their analysis stresses that scaling requires not only more neurons per chip but also distributed hierarchies, sparsity, reconfigurability, and resource awareness. This aligns with the limitations identified in Section 4.2.6., where algorithm–hardware mismatch prevents current designs from scaling efficiently. A stronger ecosystem of interoperable frameworks, common abstractions, and high-level toolchains is required to make SNN hardware accessible and portable.

### 6.2. Hardware Bottlenecks and Roofline Models

Bouvier et al. [106] provide an early survey of SNN hardware, identifying the memory bottleneck, mapping inefficiencies, and the difficulty of on-chip learning as enduring challenges. Verhelst et al. [107] extend this by applying roofline analysis to machine-learning accelerators, providing a structured framework to balance throughput, energy, and memory bandwidth. Applying such principles to SNN accelerators could address the limitations discussed in Section 4.2.5, offering a principled way to analyse energy–latency trade-offs and highlight where sparsity, quantisation, and CiM techniques offer benefits.

### 6.3. Sensor Integration and Data Bottlenecks

Delic and Afshar [108] show how SPAD-based LiDAR systems can benefit from neuromorphic event-driven processing to reduce bandwidth and noise. Their case study emphasises the importance of sensor–compute co-design, a gap also identified in Section 4.2.3. Neuromorphic extensions of SPAD arrays, ToF sensors, and other event-driven imagers remain rarely explored in dedicated hardware implementations outside of classification tasks, highlighting a rich avenue for future research in imaging pipelines.

### 6.4. Training and Algorithmic Inefficiencies

Malcolm and Casco-Rodriguez [109] review advances in training and optimisation, noting persistent challenges in ANN-SNN conversion, surrogate gradients, and convergence speed. These reinforce the limitations in Section 4.2.2, where conversion increases latency and direct training remains costly. Future directions include hardware-aware training, hybrid models, and biologically inspired local learning rules that can bridge algorithmic efficiency with physical feasibility.

### 6.5. Applications Beyond Vision

Sabbella et al. [110] survey SNNs for ubiquitous computing, highlighting applications in health, wearables, and Internet-of-Things (IoT) that predominantly involve time series sensor data. While their focus is not imaging, their work illustrates how SNNs can support a wider range of sensing modalities beyond static visual classification. In contrast, the analysis in Section 4.2.4. shows that imaging applications remain strongly biased toward classification and regression tasks, with limited exploration of segmentation or multimodal integration. Lessons from ubiquitous computing—such as energy-efficient encoding strategies and lightweight recurrent SNNs—could be adopted in future imaging models that extend beyond classification-focused pipelines.

### 6.6. Standardisation and Benchmarks

Across all reviews, a recurring theme is the lack of common evaluation procedures. Kudithipudi et al. [105] stress the need for ecosystem-level standards, while Sabbella et al. [110] propose updated software/hardware taxonomies for reproducibility. This aligns directly with Section 4.2.7, where inconsistent reporting does not facilitate comparison. Establishing standardised benchmarks for energy, latency, and throughput across both algorithmic and hardware platforms is a prerequisite for credible progress.

### 6.7. Summary

Together, these reviews converge on a picture of a field that has advanced significantly in simulation and niche hardware prototypes but faces systemic challenges in scaling, integration, and standardisation. Linking back to the limitations identified in Section 4, future progress will require: (i) ecosystem-level toolchains that abstract hardware diversity, (ii) evaluation using roofline and benchmark frameworks, (iii) sensor–network co-design for data efficiency, and (iv) expansion of applications beyond classification. These directions mark the path towards practical, generalisable, and energy-efficient SNN deployment in real-world imaging systems.

## 7. Conclusions

This study has presented a comprehensive review and comparative analysis of SNNs for imaging applications, with a particular focus on the integration of sensors, algorithms, and hardware platforms. Collecting results from a wide range of studies, several consistent patterns and limitations were identified.

### 7.1. Summary of Findings

The comparative analysis highlighted the following:

- Application bias: Most of the studies focus on classification and regression tasks, with segmentation and reconstruction being far less represented.
- Dataset limitations: Research is dominated by small, constrained, or custom datasets (e.g., MNIST, CIFAR-10, DVS Gesture), raising concerns about robustness and generalisation to high-resolution, real-world imaging.
- Training inefficiencies: ANN-SNN conversion remains the most widely adopted training strategy, but it often increases latency and energy consumption. Direct training and hybrid methods show promise but are computationally expensive and under-explored at scale.
- Hardware gap: Most evaluations remain at the CPU/GPU simulation level. Dedicated FPGA, ASIC, or neuromorphic processor implementations are comparatively rare.
- Energy and latency trade-offs: Compact models achieve efficiency at the expense of accuracy, while large models scale poorly in energy and latency. Reported efficiency gains are often based on estimates rather than measured benchmarks.
- Sensor–network co-design: Few works explicitly optimise sensor design with network architectures. Novel sensors such ToF devices remain largely unexplored in hardware-integrated SNN pipelines.
- Inconsistent reporting: Lack of standardised benchmarks for energy, latency, and throughput hampers fair comparison and slows progress.

### 7.2. Outlook

While Section 6 provided a detailed discussion of challenges and future directions, the key insights emerging from this review can be distilled into three priorities: the establishment of standardised benchmarks for reproducibility, the adoption of hardware-aware training and co-design practices, and the expansion of SNN applications beyond classification into more complex imaging tasks. Together, these steps represent essential conditions for translating current research progress into practical deployments.

To allow for fair comparison between future publications, it is recommended for authors to report comparable figures of merits such as latency at a specific resolution, number of parameters, and energy per inference to enable more consistent benchmarking across studies. Existing neuromorphic datasets, such as N-MNIST and DVS128-Gesture, are primarily based on intensity changes, but there remains a lack of single-photon inference. Although recent efforts have introduced synthetic single-photon datasets [111], these are still intensity-based rather than time-resolved, limiting their suitability for event-driven depth or photon-level tasks. This limitation motivated the use of synthetic training data for the case study rather than real-world measurements.

The case study further illustrated the promise of implementing LMU-based depth estimation in dToF applications due to their robustness, energy efficiency, and low model complexity. A natural next step is to efficiently map such networks onto hardware, optimise their architectures for improved precision, and extend their use to tasks beyond regression.

### 7.3. Closing Remarks

Spiking neural networks hold significant promise for enabling energy-efficient, event-driven computation in imaging, but the field is still at an early stage. Current progress is dominated by simulation-based classification studies, which demonstrate feasibility but not real-world readiness. Moving forward, progress will depend on bridging gaps in datasets, training, hardware, and benchmarks while embracing sensor, network, and hardware co-design. Addressing these could allow SNNs to evolve from a promising research direction into an efficient technology for next-generation imaging systems across embedded, scientific, and medical domains.

## Figures and Tables

**Table 1:** Summary of representative sensor types, outlining their typical pre-processing steps and example operations used in SNN-based imaging pipelines.

Sensor Type	Output	Pre-Processing	Example Operations	Example Works
Event-based (DVS, Spike)	Asynchronous events	Temporal Structuring	Fixed-window event binning, voxel grid	[25,26,27,29,30,32,35]
		Event Noise Filtering	Thresholding	
		Signal Normalisation	Polarity	
ToF/LiDAR	Depth image	Temporal Structuring	Voxel grid	[37,38,39]
		Event Noise Filtering	Histogram	
SPAD	Photon arrival timestamps	Temporal Structuring	Photon arrival time encoding	[42,44,45]
	Intensity	Event Noise Filtering	Spatio-temporal filters, histogram thresholding	
CIS	Continuous-intensity frames	Signal Normalisation	Crop, resize, scale, feature normalisation	[46]

**Table 2:** Summary of hardware platforms reported in the literature, detailing their architecture, number of cores, on-chip memory, power consumption technology node, and key features.

Platform	Architecture	Core Count/Scale	On-Chip Memory (MB)	Power Consumption (W) *^1^	Technology Node (nm)	Key Features	Example Works
Loihi 1/2 (Intel)	Digital Neuromorphic	128 Neuro-cores	33/37 *^2^	1.5/1	14/4	On-chip learning, graded spikes, programmable neurons, mixed-precision synapses, flexible memory allocation	[65,82]
TrueNorth (IBM)	Digital Neuromorphic	4096 cores	53 *^3^	0.3	28	Binary spikes, fixed-weight synapses	[13]
SpiNNaker (Manchester University)	Digital Neuromorphic	Up to 1M cores	128	1	130	Many-core ARM-based system, asynchronous routing	[14]
FPGA	Reconfigurable Digital	Model dependent	2–20 *^4^	0.7–4	16–28 *^4^	Reconfigurable logic, hardware-level flexibility, quick prototyping, sensor co-design capability	[32,46,86,87]
ASIC	Analogue/Digital/Mixed Signal	Custom	0.5	0.9	55,180	Fully custom design, CiM achieves very low power and fast inference	[44,45]
Memristive	Analogue/Mixed Signal	Array-Based	In-memory crossbar	0.003	65	CiM, collocated memory and computation for ultra-low-energy processing	[41]

*^1^ As reported. *^2^ Estimated from 128 neuro-cores with 192 KB/core plus 2 MB/processor. *^3^ Estimated from 4096 cores with 256 rows and 410 columns of Static Random Access Memory (SRAM) = 13 KB/core, resulting in 53 MB total. *^4^ From AMD FPGAs product family overview and datasheets [93].
